# Long COVID involves activation of proinflammatory and immune exhaustion pathways

**DOI:** 10.1038/s41590-025-02353-x

**Published:** 2025-12-12

**Authors:** Malika Aid, Valentin Boero-Teyssier, Katherine McMahan, Rammy Dang, Michael Doyle, Nazim Belabbaci, Erica Borducchi, Ai-ris Y. Collier, Janet Mullington, Dan H. Barouch

**Affiliations:** 1 Center for Virology and Vaccine Research, Beth Israel Deaconess Medical Center, Boston, MA USA; 2 Division of Sleep Medicine, Beth Israel Deaconess Medical Center, Boston, MA USA

**Keywords:** Viral infection, SARS-CoV-2

## Abstract

Long COVID (LC) involves a spectrum of chronic symptoms after acute severe acute respiratory syndrome coronavirus 2 infection. Current hypotheses for the pathogenesis of LC include persistent virus, tissue damage, autoimmunity, endocrine insufficiency, immune dysfunction and complement activation. We performed immunological, virological, transcriptomic and proteomic analyses from a cohort of 142 individuals between 2020 and 2021, including uninfected controls (*n* = 35), acutely infected individuals (*n* = 54), convalescent controls (*n* = 24) and patients with LC (*n* = 28). The LC group was characterized by persistent immune activation and proinflammatory responses for more than 180 days after initial infection compared with convalescent controls, including upregulation of JAK-STAT, interleukin-6, complement, metabolism and T cell exhaustion pathways. Similar findings were observed in a second cohort enrolled between 2023 and 2024, including convalescent controls (*n* = 20) and patients with LC (*n* = 18). These data suggest that LC is characterized by persistent activation of chronic inflammatory pathways, suggesting new therapeutic targets and potential biomarkers of disease.

## Abstract (web summary)

Long COVID (LC) involves a spectrum of chronic symptoms after resolution of acute severe acute respiratory syndrome coronavirus 2 infection. Barouch and colleagues show that LC is characterized by persistent activation of chronic inflammatory pathways and T cell exhaustion.

## Main

Long COVID (LC), also known as post-acute sequelae of coronavirus disease 2019 (COVID-19) (PASC) or post-COVID-19 condition (PCC), is characterized by multi-organ symptoms that can persist for months or years after recovery from acute COVID-19 infection^1–5^. LC prevalence estimates vary widely; some estimates of the percentage of those infected with COVID-19 who develop LC are more than 10% (ref. ^2^). Risk factors for LC include the severity of the acute infection, age, sex and preexisting health conditions; the most common symptoms are fatigue, brain fog, exercise intolerance and cognitive impairment^4,6^.

The pathophysiology of LC^1,7–11^ remains unclear but may involve increased complement activation, metabolomic abnormalities, endocrine insufficiency, inflammatory responses and uncoordinated immune responses^3,9,10,12–19^. Current hypotheses include persistent severe acute respiratory syndrome coronavirus 2 (SARS-CoV-2) or viral remnants^20,21^, autoimmunity^4,7,22,23^, cortisol insufficiency^12,24^, latent herpesvirus reactivation^25^, metabolic dysfunction^19,22,26,27^, T cell dysregulation^28^ and inflammatory tissue damage^2,6,9,14,16,29–33^. Given the diversity of signs and symptoms of LC, treatment is typically symptomatic and personalized, with an emphasis on rehabilitation. The STOP-PASC trial^34^ revealed that a 15-day course of nirmatrelvir-ritonavir showed no significant improvement in treating LC (PASC) symptoms such as fatigue, brain fog and shortness of breath^34^, underlining the need for new therapeutic approaches for LC.

In this study, we evaluated the immunological and inflammatory responses in people with LC compared with convalescent controls (CCs) at 90–180 days and more than 180 days after initial COVID-19 infection using immunological assays, virological assays, transcriptomics and proteomics. The first cohort was enrolled in 2020–2021, and the second cohort was enrolled in 2023–2024. Our data show that chronic inflammation, T cell exhaustion, metabolic dysregulation and upregulation of the JAK-STAT and interleukin-6 (IL-6) signaling pathways are key features of LC.

## Results

### Proinflammatory pathways are persistently upregulated in LC

**Fig. 1:**
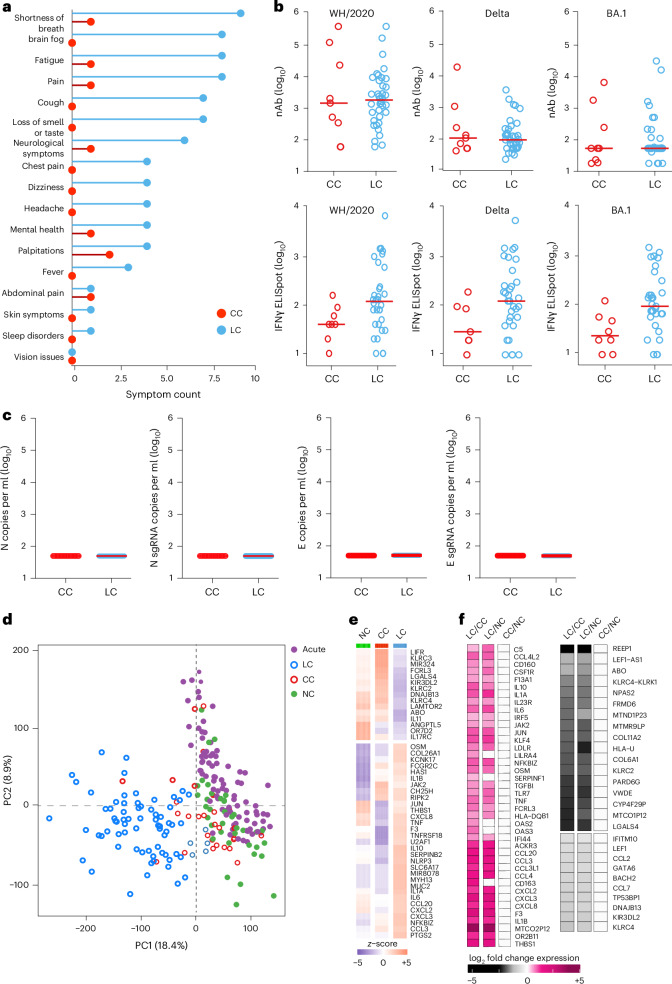
Immunological and virological profiling of the 2020–2021 cohort. **a**, Symptom prevalence in individuals with LC (*n* = 28) and individuals who recovered (CCs, *n* = 24) in the 2020–2021 cohort. **b**, log_10_-transformed nAbs titers and IFNγ (ELISpot responses against the WH/2020, Delta and BA.1 variants of SARS-CoV-2) in CC (*n* = 9) and LC (*n* = 41) individuals on day 90–180 after infection. The dots are individual participants and the red bars are group medians. Samples from several time points were assessed for participants with LC. **c**, Quantification of SARS-CoV-2 genomic (N, E) and subgenomic (N single-guide RNA (sgRNA), E sgRNA) RNA in CCs (*n* = 9) and LCs (*n* = 41). Medians (red bars) are shown for each group. Samples from several time points were assessed for participants with LC. **d**, *K*-mean clustering across acute COVID-19 (*n* = 54), LCs (*n* = 28), CCs (*n* = 24) and uninfected (NC) (*n* = 35) individuals. All available samples were included in the *k*-mean analysis for the LC and CC groups. **e**, Heatmap of top significant (*P*_adj_ < 0.05) proinflammatory genes upregulated (red) or downregulated (blue) in LCs compared to CCs or NCs at day 90–180 after SARS-CoV-2 infection. **f**, Transformed log_2_ fold change expression of the top upregulated (pink) and downregulated (dark) genes in LCs compared to CCs and NCs (*P*_adj_ < 0.05).

We evaluated samples from 142 participants who were enrolled in an observational cohort of the Massachusetts Consortium for Pathogen Readiness (MassCPR) and Beth Israel Deaconess Medical Center (BIDMC) in Boston between April 2020 and October 2021 (hereafter the 2020–2021 cohort). This cohort included UCs (uninfected; *n* = 35), acutely infected individuals less than 30 days after COVID-19 infection (acute; *n* = 54), CCs (*n* = 24) and patients with LC (*n* = 28) (Table 1 and Supplementary Table 1). Clinical symptoms in the LC group included primarily shortness of breath, brain fog, fatigue, pain, cough and abnormal smell and taste (Fig. 1a), which is consistent with prior reports^9,16,23,30,35–40^. Peripheral blood mononuclear cells (PBMCs) were collected during the acute phase (<30 days) (LC: *n* = 6; CC: *n* = 5), 90–180 days (LC: *n* = 26; CC: *n* = 21) and more than 180 days (LC: *n* = 21; CC: *n* = 5) after SARS-CoV-2 infection. Plasma samples were also collected during the acute phase (LC: *n* = 8; CC: *n* = 4), 90–180 days (LC: *n* = 22; CC: *n* = 8) and more than 180 days (LC: *n* = 19; CC: *n* = 6) after SARS-CoV-2 infection. We performed immunological and virological assays, bulk RNA sequencing (RNA-seq) and plasma proteomics.

**Table 1:** Description and demographics for the 2020–2021 cohort

	NCs (*n* = 35)	Acute COVID 19^+^ infection (*n* = 54)	CCs (*n* = 24)	LCs (*n* = 28)
Age	Median: 63	Median: 65.75	Median: 50.05	Median: 50.50
	95% CI	95% CI	95% CI	95% CI
	(54.39–66.71)	(59.57–69.07)	(46.28–59.21)	(45–56.15)
Gender				
Male	18 (51.42%)	27 (50%)	10 (40%)	4 (14.29%)
Female	17 (48.57%)	27 (50%)	15 (60%)	24 (85.71%)
Ethnicity				
Asian	2 (5.71%)	ND	0 (0%)	ND
Black	6 (17.14%)	ND	5 (20%)	ND
White	22 (62.85%)	ND	11 (44%)	ND
Hispanic	5 (14.28%)	ND	2 (8%)	ND
Not Hispanic or Latino	0 (0%)	ND	7 (28%)	28 (100%)
Number of days to first COVID^+^	NA	Median: 14	Median: 71	Median: 200
		95% CI	95% CI	95% CI
		(11.6–16.40)	(44.67–97.33)	(178.27–221.73)
Vaccine	NA	NA		
Moderna			3 (12%)	12 (42.85%)
Pfizer			2 (8%)	12 (42.85%)
Unknown			2 (8%)	4 (14.28%)
No vaccine			18 (72%)	0 (0%)

NA, not applicable; ND, unknown.

**Extended Data Fig. 1:**
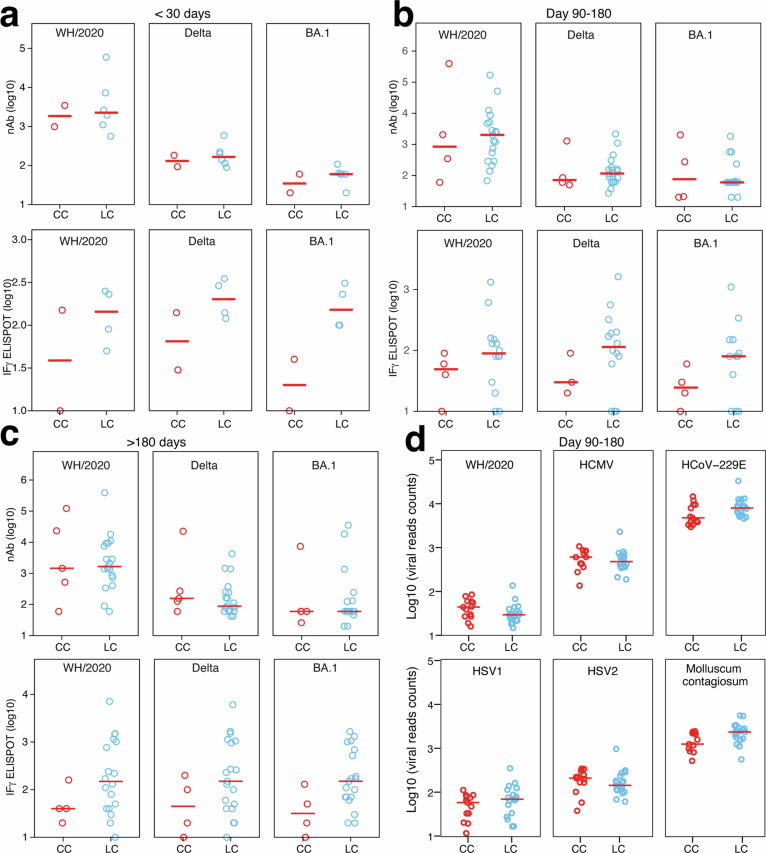
Immunologic and virologic profiling in the 2020/2021 cohort. **a**-**c**, Neutralizing antibody (NAb) titers and pooled peptide Spike-specific IFN-g responses against SARS-CoV-2 WA1/2020, Delta and BA.1 at < 30 days (**a**), 90-180 days (**b**), and >180 days (**c**) after SARS-CoV-2 infection in the CC and the LC groups. **d**, Viral reads count for multiple viruses in the CC and LC groups. Group comparison is performed using the two-sided Mann-Whitney tests. Medians (red bars) shown for each group. For all plots P > 0.05 is considered not significant.

Assessment of SARS-CoV-2 neutralizing antibody (nAb) responses using luciferase (LUC)-based pseudovirus neutralization assays and T cell responses using pooled peptide interferon-γ (IFNγ) enzyme-linked immunospot (ELISpot) assays against SARS-CoV-2 WA1/2020, Delta and Omicron BA.1 in the CC and the LC groups did not detect differences in SARS-CoV-2 nAb titers and identified a trend toward higher Spike-specific IFNγ ELISpot responses in the LC compared with the CC group (Fig. 1b and Extended Data Fig. 1a–c). We did not detect plasma SARS-CoV-2 viral loads in any CCs or individuals with LC using PCR with reverse transcription genomic or subgenomic viral load assays (Fig. 1c).

Bulk RNA-seq in PBMCs from individuals with LC (*n* = 26) and CCs (*n* = 21) at day 90–180 and in uninfected (*n* = 35) and acute (*n* = 54) individuals identified reads that mapped to the human genome and multiple common viruses (SARS-CoV-2, varicella zoster virus, Western equine encephalitis virus, Epstein–Barr virus, human cytomegalovirus, herpes simplex virus 1 and 2, influenza virus, rotavirus). We did not detect significant differences in viral read counts for these common viruses in the LC compared with the CC group (Extended Data Fig. 1d). Unsupervised clustering of bulk RNA-seq transcriptomic data revealed a distinct stratification between the LC group and the other cohorts, whereas the CC group clustered with the UCs (Fig. 1d). Differential bulk RNA-seq gene expression analysis in PBMCs across these groups showed upregulation of multiple proinflammatory markers in the LC compared with the CC group and uninfected individuals, including chemokines and cytokines (*CXCL2*, *CXCL3*, *CCL3*, *IL10*, *IFNG*, *IL6*, *TNF*, *IL1B*, *IL1A*, *NFKBIZ*), the *NLRP3* inflammasome and the complement and coagulation genes *C5*, *F3* and *THBS1* (Fig. 1e,f). In contrast, downregulation of activating (for example, *KLRC2*) and inhibitory (for example, *KLRC1*, *KIR3DL2*) natural killer (NK) cell receptors and T cell activation markers in the LC compared to the CC group was observed (Fig. 1f).

**Fig. 2:**
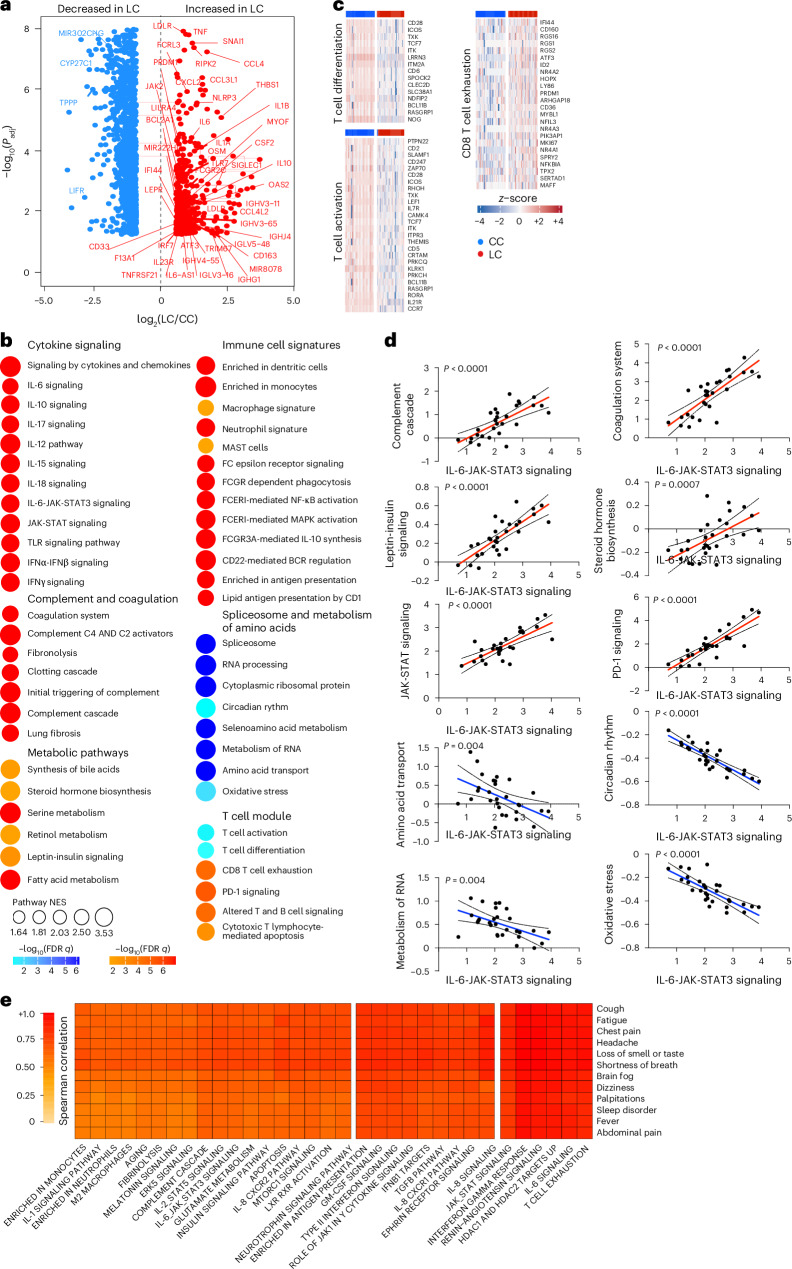
Transcriptomic differences between LC and CC groups in the 2020–2021 cohort. **a**, Scatter plot of the upregulated (red) or downregulated (blue) genes in the LC group (*n* = 26) compared with the CC (*n* = 21) group at day 90–180 after infection. **b**, Dot plots of the pathway normalized enrichment scores (NES) in LC compared with CC at day 90–180 after infection. The dot size illustrates the normalized enrichment scores. The color gradients reflect the GSEA (FDR) *q* > 0.05. **c**, Heatmaps of the normalized *z*-score gene expression of the top markers in the LC and CC group level for pathways of T cell activation, T cell differentiation and CD8^+^ T cell exhaustion. **d**, Correlation of the IL-6-JAK-STAT3 signaling pathways with pathways upregulated or downregulated in the LC group. The red and blue lines indicate linear regression. The gray shading represents the 95% confidence interval (CI). *P* values are indicated. **e**, Heatmap of Spearman correlations between clinical symptoms and pathway activity levels in the LC group at day 90–180 after infection.

**Extended Data Fig. 2:**
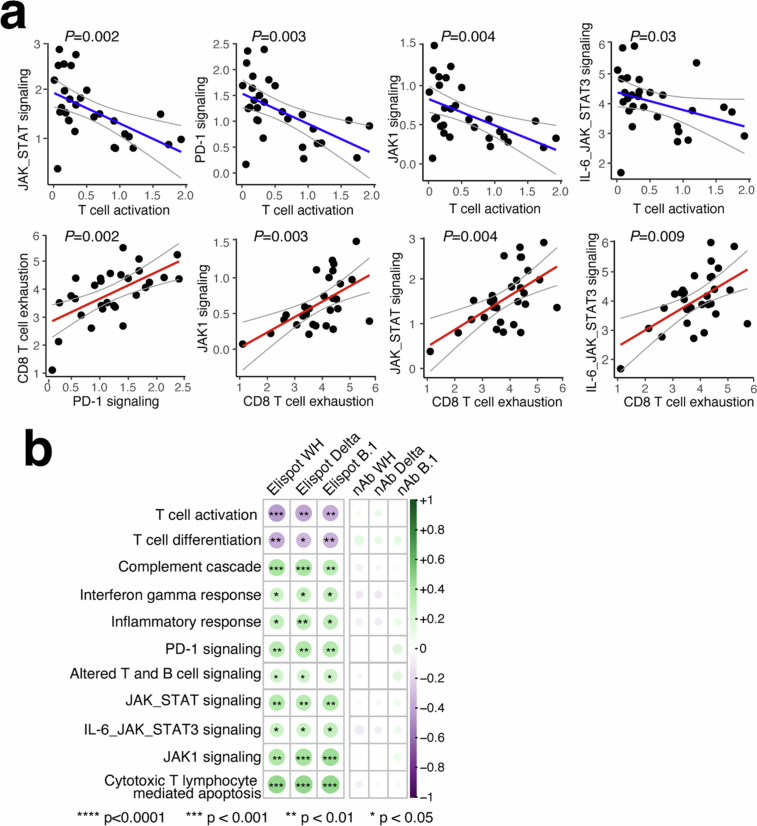
T cell dysfunction in the LC group. **a**, Scatter plots showing the correlation of T cell activation, PD-1 signaling and CD8 T cell exhaustion with JAK_STAT and JAK1 signaling pathways in the LC group at 90-180 days after infection. The gray shadow covers the 95% confidence interval. **b**, Correlation plot between T cell responses and neutralizing titers for WH, Delta and Omicron variants and top pathways in the LC group. Circles show Spearman rank correlations (ρ); size scales with |ρ| and color encodes the two-sided t test p value. * p < 0.05, ** p < 0.01, *** p < 0.001.

Analysis of differentially expressed genes in the LC group compared with the CC group at day 90–180 after infection identified an increase of multiple proinflammatory markers, such as *IL6*, *NLRP3*, *TNF*, *JAK2*, *CSF2*, *IL1B* and *IL10*, in the LC compared with the CC group (Fig. 2a). Pathway enrichment analysis revealed upregulation of signatures associated with signaling by proinflammatory cytokines such as IL-6, IFNα, IFNβ and IFNγ, JAK-STAT pathways, complement and coagulation cascade, metabolic pathways and immune cell signatures of monocytes, macrophages, neutrophils and dendritic cells (Fig. 2b), while RNA processing and nitrogen metabolism, oxidative stress and amino acid transport, were decreased in the LC compared with the CC group (Fig. 2b). Additionally, transcriptomic signatures of T cell activation and differentiation (*CD28*, *ICOS*, *TCF7*) were downregulated in the LC compared with the CC group at day 90–180 after infection (Fig. 2b), while CD8^+^ T cell exhaustion signatures and programmed cell death protein 1 (*PDCD1*) signaling-associated genes (*IFI44*, *PRDM1*, *NR4A3*, *NFKBIA*, *MAFF*) were significantly increased in the LC group (Fig. 2c), suggesting a potential role of T cell dysregulation in the pathogenesis of LC. Moreover, JAK1, JAK-STAT and IL-6 signaling pathways correlated inversely with T cell activation and positively with CD8^+^ T cell exhaustion and PD-1 signaling (Extended Data Fig. 2a). Signatures of T cell activation and differentiation were positively correlated with IFNγ ELISpot responses, whereas proinflammatory signaling and immune exhaustion signatures were negatively correlated with IFNγ ELISpot responses (Extended Data Fig. 2b). We observed a significant correlation between IL-6 and JAK-STAT signaling pathways with complement and coagulation pathways, metabolic signatures and PD-1 signaling in the LC group (Fig. 2d), suggesting a potentially coordinated role of these pathways in the pathogenesis of LC, while the IL-6 and JAK-STAT signaling pathways correlated negatively with the metabolism of amino acids and oxidative stress in the LC group (Fig. 2d). IFNγ, IL-6, JAK-STAT and T cell exhaustion pathways correlated with clinical symptoms in the group with LC, including fatigue, shortness of breath and cognitive complaints (Fig. 2e).

**Fig. 3:**
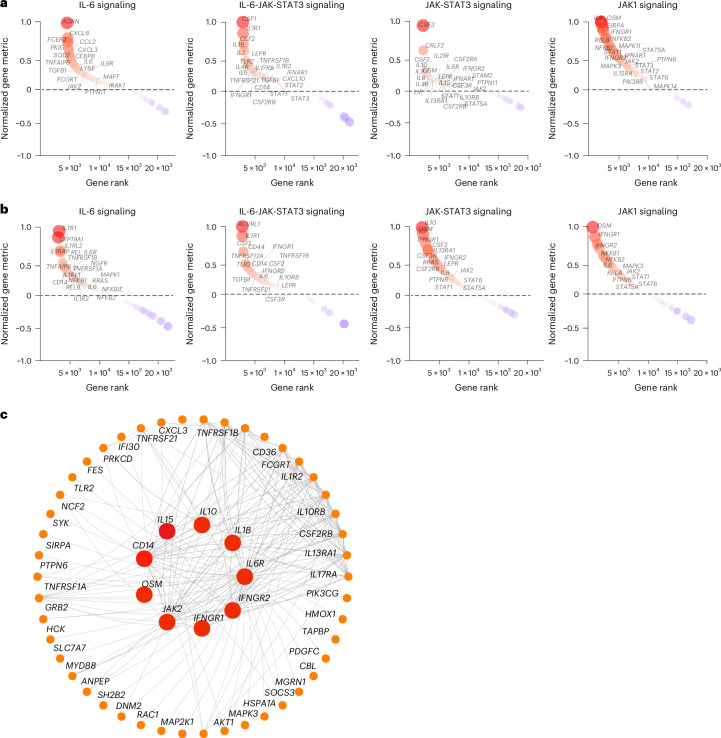
Persistent activation of IL-6 and JAK-STAT signaling pathways in the 2020–2021 cohort. **a**,**b**, Upregulated IL-6, IL-6-JAK-STAT3, JAK-STAT and JAK1 signaling pathways at day 90–180 (**a**) and more than 180 days (**b**) after SARS-CoV-2 infection in the LC group compared with the CC group. The top genes driving the enrichment of each pathway are shown in the red circles. The *x* axis represents each gene’s rank in the gene list, while the *y* axis shows the corresponding gene rank metric score. **c**, Gene interaction network analysis showing the top leading genes for the IL-6 and JAK-STAT signaling pathways. The edges represent the Spearman correlation between genes (*P* < 0.05). Hub genes with a high number of correlated genes are represented by larger circles.

**Extended Data Fig. 3:**
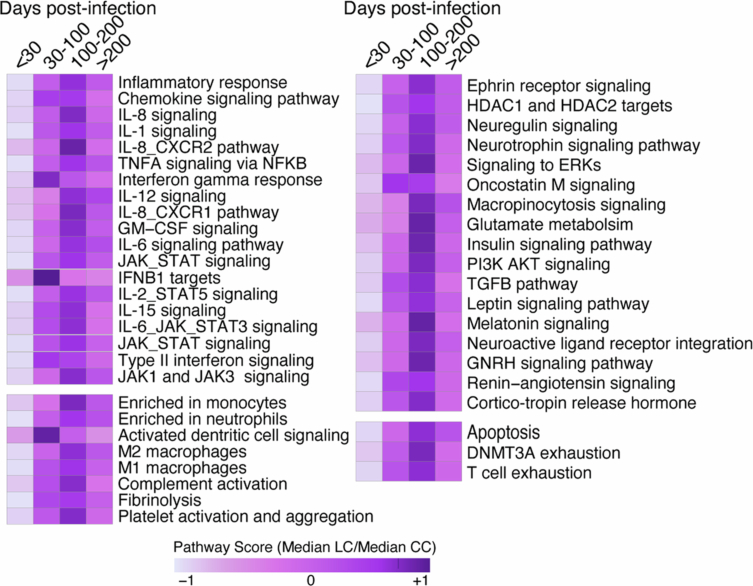
Persistent proinflammatory pathways in the 2020/2021 cohort. Heatmap showing the median ratio ssGSEA score shown as the LC/CC ratio (row-normalized) for each pathway at <30 days (LC, *N* = 6; CC, *N* = 5), 30-100 days (LC, *N* = 7; CC, *N* = 4), 100-200 days (LC, *N* = 22; CC, *N* = 5) and 200-365 days (LC, *N* = 22; CC, *N* = 6).

To investigate the timing of the chronic inflammatory pathways in the LC group in finer detail, we performed gene set enrichment analysis (GSEA) using blood samples collected at less than 30 days (LC, *n* = 6; CC, *n* = 5), day 30–100 (LC, *n* = 7; CC, *n* = 4), day 100–200 (LC, *n* = 22; CC, *n* = 5) and day 200–300 (LC, *n* = 22; CC, *n* = 6) after infection from individuals with LC and CCs. We observed upregulation of proinflammatory pathways associated with IL-1, IL-6, JAK-STAT, IFN signaling, cell cycle, metabolic pathways, complement activation and T cell exhaustion pathways in the LC group by day 30, with increased and sustained effects for over 200 days (Extended Data Fig. 3). Pathways associated with IL-6, JAK-STAT and JAK1 signaling were upregulated at both day 90–180 (Fig. 3a) and more than 180 days (Fig. 3b) in the LC group compared to the CC group, as highlighted by the persistent increase of several leading genes in the pathways, including *IL6R*, *IL6*, *IL1R*, *CD14*, *CSF1*, *CSF3RLEPR*, *IL4R*, *STAT1*, *STAT3* and *JAK2* (Fig. 3a,b). Weighted gene correlation network analysis revealed that individual markers of the JAK-STAT and IL-6 signaling pathways, including *IL6R*, *AKT1*, *JAK2*, *IL1B*, *IFNGR1* and *IFNGR2* and *CD14* were highly correlated and positively associated with the subsequent development of LC (Fig. 3c).

**Fig. 4:**
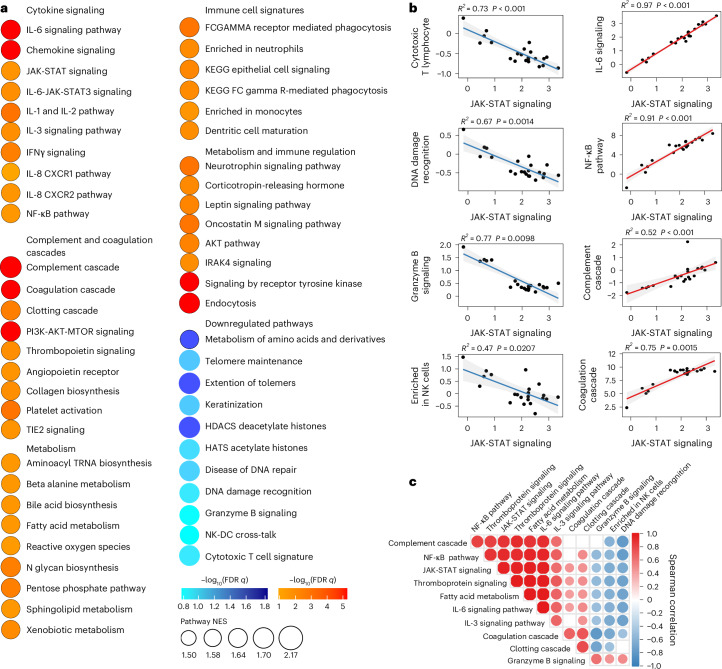
Proteomic differences between LC and CC groups in the 2020–2021 cohort. **a**, Dot plots representing pathway NES scores (GSEA, nominal *P* < 0.05) in LC (*n* = 22) versus CC (*n* = 8) at day 90–180 after infection. Upregulated pathways are shown in red gradient and downregulated pathways are shown in blue gradient. Color gradients reflect a GSEA *P* < 0.0.5. **b**, Correlation of JAK-STAT signaling and selected pathways from **a**. Each point represents the per-patient single-sample GSEA (ssGSEA) enrichment score for the two indicated pathways. The lines show the ordinary least-squares fit with shaded 95% CIs. The Spearman correlation linear model *R*^2^ and two-sided *P* values are annotated. The red and blue fits indicate positive and negative slopes. **c**, Pathway Spearman correlation matrix between pathways upregulated or downregulated in the LC group compared with the CC group at 90–180 days after SARS-CoV-2 infection. The circle color encodes the sign of the Spearman correlation coefficient *ρ* (red, positive; blue, negative) and circle size encodes |*ρ* | ; nonsignificant correlations (*P* > 0.05) are shown as empty squares.

We also performed proteomics analysis on plasma from the LC (*n* = 22) and CC (*n* = 8) groups at day 90–180 (Fig. 4a and Supplementary Table 2). We observed increased levels of plasma cytokine signaling, including JAK-STAT (STAT5, STAT1, IL6ST, SOS1, RELB), IL-6, NF-κB signaling, complement and coagulation cascades (TMPRSS6, F8, C9, C1S, F9, C6, C1R, FN1), metabolic pathways (PTPN11, LEPR, PTEN, EIF4E) and corticotropin-releasing hormone and leptin signaling (TCF4, PRKCA, PRKCB, PLCG1) in the LC group compared to the CC group at day 90–180 after infection (Fig. 4a), while DNA damage repair (RAD51C, RAD51D, HUS1, MSH2, RPA2, PARP1, YY1, CETN2), cytotoxic T cell (DFFA, BCL2, FADD, B2M, CD3G, HLA-E, CD247, HLA-G, BID), telomere maintenance (PCNA, PRIM1, TEN1, RUVBL1, RPA2) and amino acid metabolism (GNMT, PDHB, DLD, AMT, PDHA2, BHMT2, GOT1, MRI1, GOT2, MTAP, CTH) were decreased in the LC group compared to the CC group at day 90–180 after infection (Fig. 4a). Furthermore, the plasma JAK-STAT signaling pathways by proteomics correlated positively with plasma proinflammatory signatures such as the NF-κB signaling pathway, complement activation, leptin signaling and corticotropin-releasing hormone signaling, and correlated negatively with plasma DNA damage repair, cytotoxic T cell and granzyme B signaling in the LC group (Fig. 4b,c). These data indicated that LC is associated with chronic inflammation, as well as immunological and metabolic dysregulation.

### Inflammation during acute infection predicts the development of LC

**Extended Data Fig. 4:**
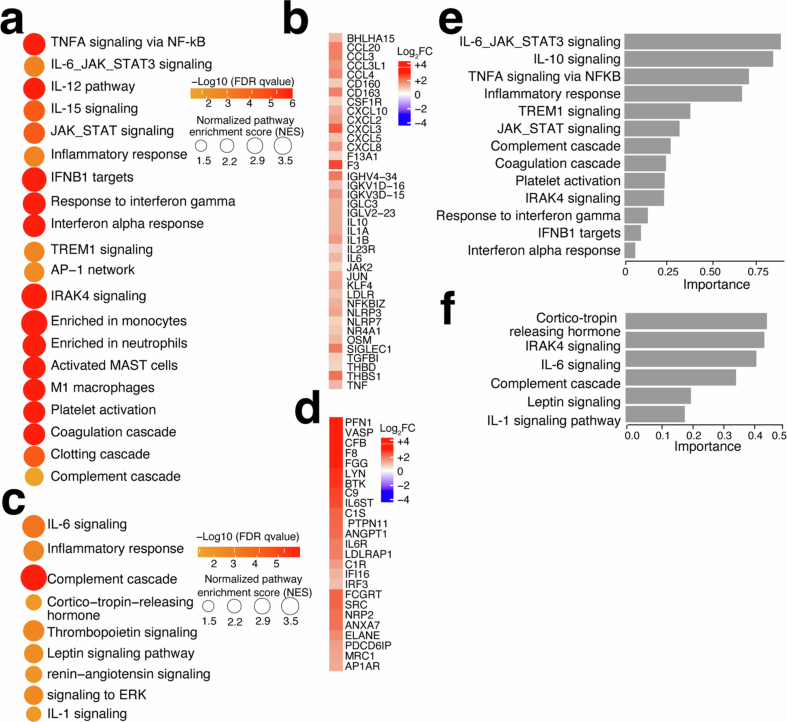
Acute inflammation is associated with long Covid in the 2020/2021 cohort. **a**-**b**, Dot plots showing the upregulated enriched blood transcriptomic pathways (**a**) and genes (**b**) increased in the LC group compared to CC at < 30 days post infection. **c**-**d**, Same as **a**-**b** for plasma proteomics pathways (**c**) and plasma markers (**d**). **e**, Bar plot showing the top key transcriptomic pathways identified by random forest analysis. The pathways are ranked in descending order of importance with respect to the accuracy of the model. **f**, same as e for the top key proteomics pathways.

To evaluate whether activation of proinflammatory pathways during acute COVID-19 infection correlated with the subsequent development of LC, we performed an exploratory analysis of transcriptomic and proteomic changes in the subset of participants in this cohort (LC: *n* = 8; CC: *n* = 5) who had PBMC and plasma samples both during acute COVID-19 (< 30 days) and at day 90–180 after infection. Transcriptomic profiling of PBMCs indicated that acutely infected participants who subsequently developed LC had higher levels of proinflammatory pathways such as IFNβ and IFNγ, JAK-STAT and IL-6 signaling, as well as innate immune cell signatures of monocytes neutrophils and complement and coagulation cascades (*CCL3*, *CCL20*, *CD160*, *F13A1*, *F3*, *IL6*, *NR4A1*, *NLRP3*, *THBS1*) during acute infection compared with acutely infected patients who fully recovered (CCs) (Extended Data Fig. 4a,b). Plasma proteomics profiling validated the significant increase of proinflammatory pathways (IL-6 signaling, complement cascade, leptin signaling pathway) during acute infection in participants who subsequently developed LC compared to those who recovered (CC) (Extended Data Fig. 4c,d).

To explore further the potential association between early activation of proinflammatory pathways during acute infection and the subsequent development of LC, we used a supervised random forest (RF) algorithm to identify key blood and plasma features during acute infection that predict the development of LC. This model revealed that gene expression and protein levels of complement activation, proinflammatory response, JAK-STAT, IL-6, IL-6-JAK-STAT3, IFNβ and IFNγ signaling pathways during acute infection were among the top predictors for the development of LC (Extended Data Fig. 4e,f). A feature importance analysis revealed upregulation of the IL-6 and JAK-STAT signaling, corticotropin-releasing hormone, IL-10 and TNF signaling pathways during the acute phase were among the top predictors for the development of LC. Together, these observations suggested that early activation of proinflammatory pathways strongly predicted LC development based on gene and protein profiling.

### Proinflammatory pathways are upregulated in a validation cohort

**Fig. 5:**
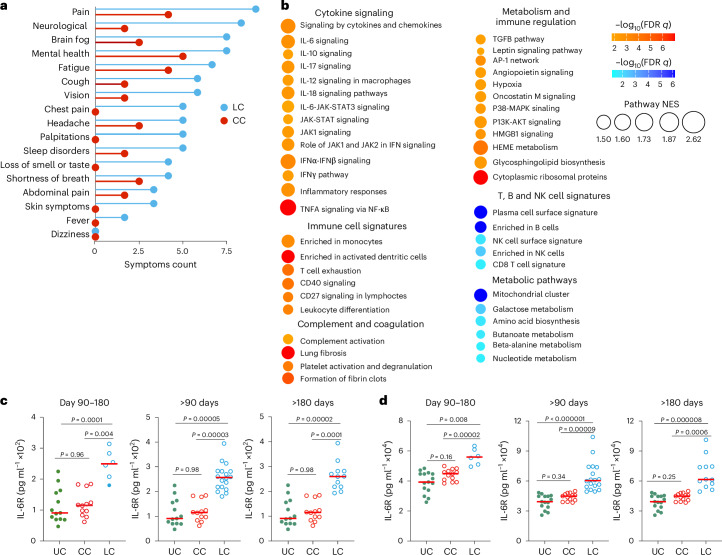
Transcriptomic differences between LC and CC groups in the 2023–2024 cohort. **a**, Symptom prevalence in individuals with LC (*n* = 18) or individuals who recovered (CC, *n* = 20) in the 2023–2024 cohort. **b**, Dot plots of NES in LC compared to CC groups at day 90–180 after SARS-CoV-2 infection. Upregulated pathways are shown in red; downregulated pathways are shown as blue gradients. Dot size indicates the NES. The color gradient indicates a GSEA FDR of *q* < 0.05. **c**,**d**, IL-6R plasma level measured using ELISA (**c**) and MSD (**d**) assays in LC (*n* = 18), CC (*n* = 13) and naive UCs (*n* = 13) at day 90–180, more than 90 days and more than 180 days after SARS-CoV-2 infection. Dots represent participants; the red horizontal bars indicate the group medians. A two-sided Kruskal–Wallis tests with post hoc pairwise comparisons using Dunn’s test with Bonferroni correction was used. *P*_adj_ values are shown.

**Extended Data Fig. 5:**
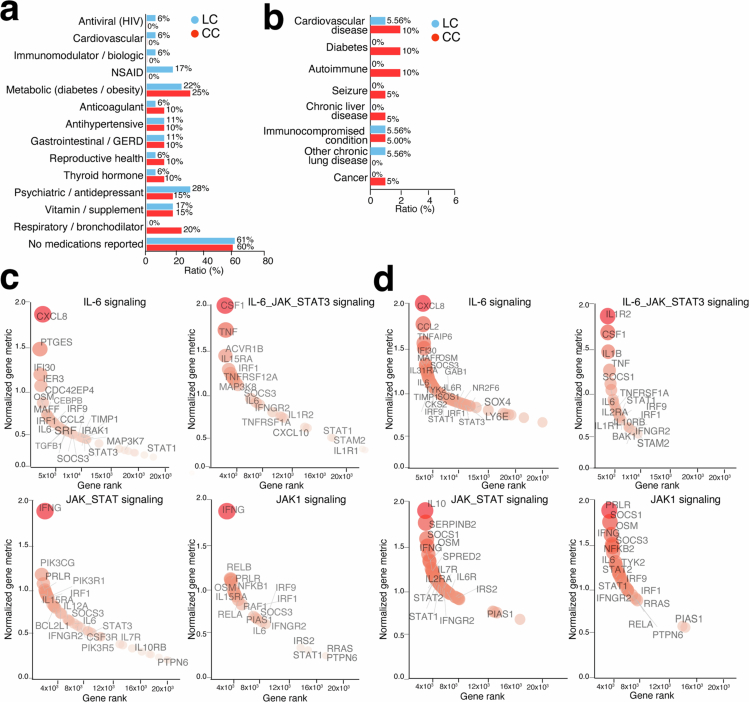
Persistent upregulation of JAK_STAT and IL-6 pathways in the 2023/2024 cohort. **a**-**b**, Bar plots showing the number (X axis) and the proportion (next to each bar) of medication history and preexisting conditions in the LC and CC groups. **c**-**d**, Rank for leading genes in the IL6, JAK_STAT and JAK1 pathways in the LC group at 90-180 (**c**) days and after 180 days (**d**) post infection. The x-axis represents each gene’s rank in the gene list, while the y-axis shows the corresponding GSEA rank metric score.

To confirm our findings in an independent cohort with LC, we performed transcriptomics profiling of peripheral blood samples collected on days 15–700 after infection from individuals with LC (*n* = 18) and CCs (*n* = 20) who were enrolled at the BIDMC clinical site of the National Institutes of Health (NIH) RECOVER prospective clinical trial between October 2022 and December 2024 (hereafter the 2023–2024 cohort) (Table 2 and Supplementary Table 3). All study participants had standardized clinical meta-data and responded to structured symptom questionnaires^41^ (Extended Data Fig. 5a,b and Supplementary Table 3). Pain, neurological symptoms, brain fog, fatigue and cough were the primary symptoms that were more frequent in the LC compared with the CC groups (Fig. 5a). Transcriptomic analysis of peripheral blood in the LC (*n* = 10) and CC (*n* = 12) groups at day 90–180 after infection indicated upregulation of pathways linked to proinflammatory cytokine signaling (IL-6, IL-10 and IL-12 signaling), complement activation, proinflammatory immune cell signaling, signatures of T cell exhaustion and certain metabolic and immune regulation pathways in the LC compared with the CC group (Fig. 5b). In contrast, pathways associated with mitochondrial function, amino acid metabolism and signatures of NK cells, T cells and B cells were downregulated in the LC compared with the CC group (Fig. 5b). In line with observations from the 2020–2021 cohort, the IL-6, JAK-STAT and JAK1 signaling pathways remained persistently upregulated in the LC group both at day 90–180 and more than 180 days compared to the CC group (Extended Data Fig. 5c,d).

**Table 2:** Description and demographics for the 2023–2024 cohort

	CCs (*n* = 20)	LCs (*n* = 18)
Age	Median: 48	Median: 55.5
	95% CI	95% CI
	(40.53–55.47)	(48.16–62.84)
Sex at birth		
Male	9 (45%)	4 (22.2%)
Female	11 (55%)	14 (77.7%)
Ethnicity		
Asian	0 (0%)	0 (0%)
Black	0 (0%)	0 (0%)
White	0 (0%)	0 (0%)
Hispanic	2 (10%)	3 (16%)
Not Hispanic or Latino	18 (90%)	15 (83%)
Race		
White	19 (95%)	15 (83%)
Native Hawaiian or other Pacific Islander	0 (0%)	1 (0.05%)
Other	0 (0%)	1 (0.05%)
Multiracial	0 (0%)	1 (0.05%)
Black or African American	1 (0.05%)	0 (0%)
Number of days to first COVID^+^	Median: 286	Median: 420
	95% CI	95% CI
	(213.63–358.37)	(239.66–600.34)
Vaccine	ND	ND
Moderna		
Pfizer		
Unknown		
No vaccine

**Extended Data Fig. 6:**
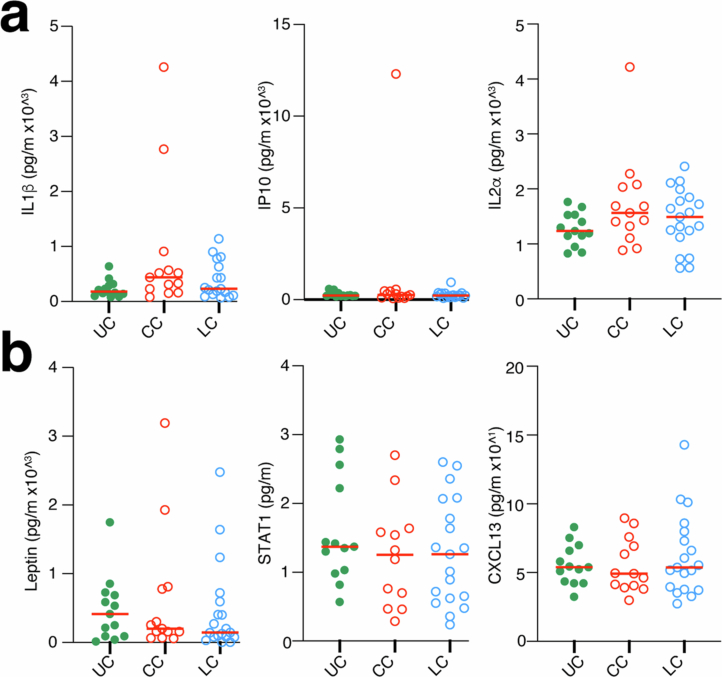
Plasma level of additional cytokines in the LC and the CC groups. **a**,**b**, Plasma level of additional cytokines measured by ELISA (**a**) and Mesoscale Discovery (MSD) (**b**) in LC (N = 19), CC (N = 13), and uninfected controls (N = 13); red horizontal bars indicate group medians; P values were assessed two-sided Kruskal–Wallis tests with post-hoc pairwise comparisons used Dunn’s test with Bonferroni correction. P > 0.05 is considered not significant.

We used enzyme-linked immunosorbent assay (ELISA) and Meso Scale Discovery (MSD) assays to evaluate the plasma levels of selected proinflammatory markers at day 90–180 (LC, *n* = 19; CC, *n* = 13), more than 180 days (LC, *n* = 19; CC, *n* = 13) and UCs (*n* = 13) in the 2023–2024 cohort (Fig. 5c,d and Extended Data Fig. 6). Plasma levels of IL-6R were significantly elevated in the LC group compared to the CC and UC groups at day 90–180 and more than 180 days using ELISA (Fig. 5c) and MSD (Fig. 5d), suggesting a link between chronic inflammation and LC^8,12,16,33^.

**Fig. 6:**
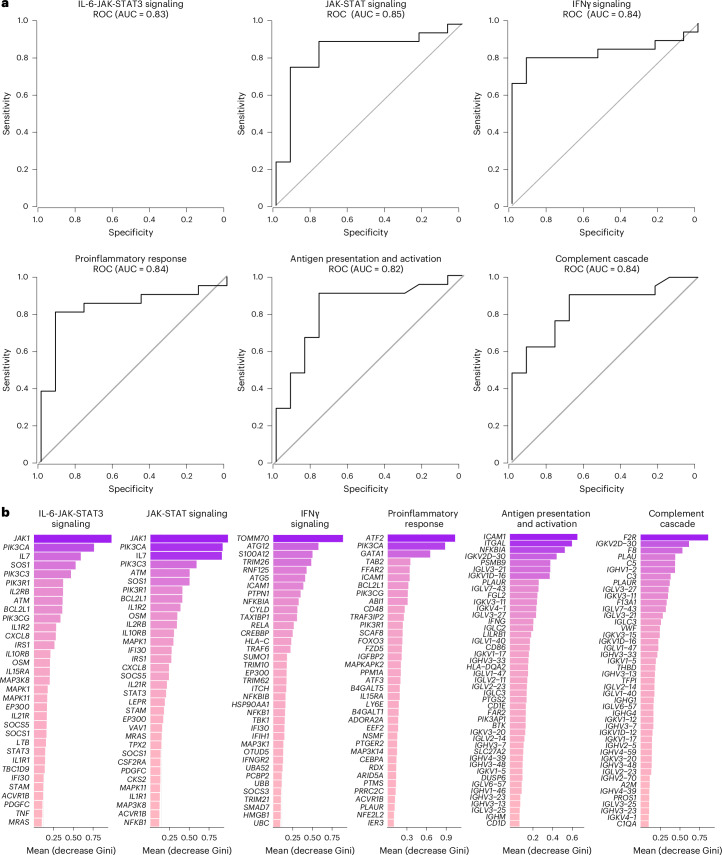
Prediction of LC status using the combined 2020–2021 and 2023–2024 cohorts. **a**, ROC curves showing the AUC from the RF analysis of individual biological pathways at day 90–180 after infection. **b**, Bar plot showing the top key genes identified using the RF analysis. The genes are ranked in descending order of importance for each pathway with respect to the accuracy of the model.

**Extended Data Fig. 7:**
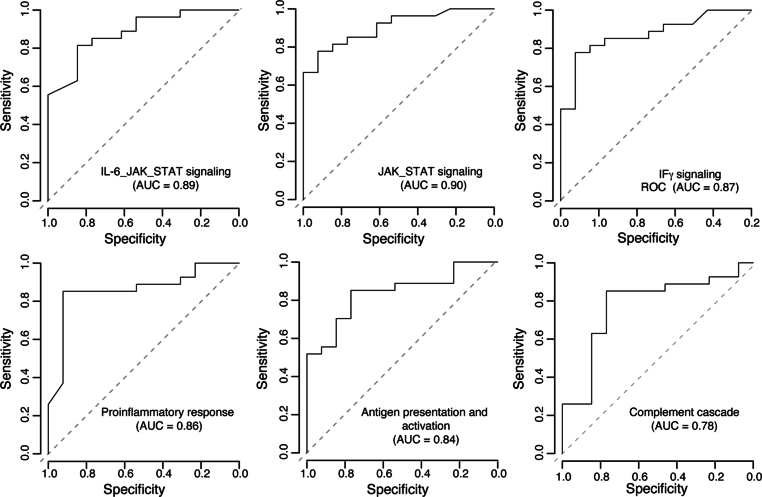
Random forest model in the combined 2020/2021 and 2023/2024 cohorts. Receiver Operating Characteristic (ROC) curves and area under the curve AUC showing the performance of random forest models trained on selected pathways at 180-365 days post infection.

We next combined data from the 2020–2021 and 2023–2024 cohorts and performed an RF analysis using the gene expression profiles at day 90–180 and day 180–365 after infection to define the pathways that correlated most robustly with LC compared with CC. For each time point, RF models were trained using gene sets corresponding to individual pathway modules, and performance was evaluated using receiver operating characteristic (ROC) curves and area under the curve (AUC) metrics. Pathways related to IL-6, JAK-STAT, IFNγ, proinflammatory response, antigen presentation and activation, and complement cascade were the top pathways associated with LC status at day 90–180 (Fig. 6a) and day 180–365 (Extended Data Fig. 7). Individual markers within the IL-6 and JAK-STAT signaling pathways, including *JAK1*, *PIK3*, *CXCL8*, *BCL2L1*, *OSM*, *MAP3K8* and *STAT3* were the top genes associated with LC (Fig. 6b).

**Extended Data Fig. 8:**
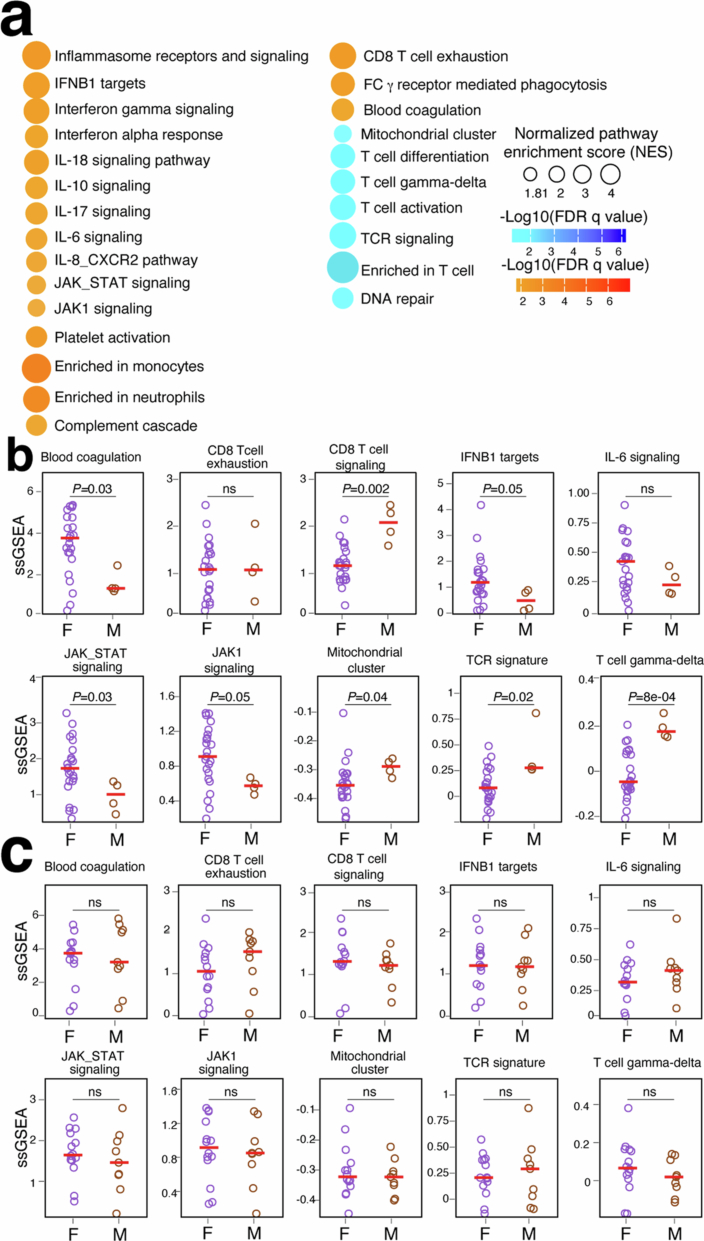
Sex differences in the combined 2020/2021 and 2023/2024 cohorts. **a**, Dot plots showing the upregulated enriched blood transcriptomic pathways upregulated (red gradient) or downregulated (blue gradient) in the female LC group (*N* = 29) compared to female CC (*N* = 14) at 90-180 days post-infection. **b**-**c**, Pathway’s sample-level score (ssGSEA) comparing female (F) to male (M) in LC (**b**) and CC (**c**) groups. Group comparison is performed using the two-sided Mann-Whitney tests. Medians (red bars) shown for each group. For all plots P > 0.05 is considered not significant (ns).

Finally, in an exploratory sex-stratified analysis at day 90–180 after infection, using transcriptomic profiling in peripheral blood in the combined cohort, females with LC (*n* = 29) showed stronger enrichment of inflammatory pathways than females who recovered (CC, *n* = 14), including monocyte signatures, proinflammatory cytokine/chemokine signaling, complement and coagulation cascades, IL-6, JAK1, JAK-STAT signaling, and T cell exhaustion (Extended Data Fig. 8a). In the LC group, these pathways were generally higher in females than males (Extended Data Fig. 8b), whereas no significant sex differences were detected within the CC group (Extended Data Fig. 8c). These data demonstrate persistent activation of proinflammatory pathways in a validation LC cohort, with possibly greater inflammation in females compared with males, although these observations require confirmation in larger studies.

## Discussion

In this study, we found that individuals with LC were characterized by persistent activation of chronic inflammatory pathways compared with CCs. These pathways included proinflammatory cytokine signaling, complement activation, metabolic dysregulation and immune exhaustion and persisted for more than 180 days. These findings suggest that chronic inflammation may contribute to the pathogenesis of LC and define potential new therapeutic targets.

We observed that participants with LC exhibited reduced granzyme B and cytotoxic T cell signaling and increased immune exhaustion, suggesting dysregulated cross-talk between the innate and adaptive immune responses^15,28,42^. Our findings are consistent with prior reports that the IL-6 and JAK-STAT signaling pathways were upregulated in individuals with LC^28,43,44^, particularly in those with cardiorespiratory or multisystem symptoms. We also found that chronic upregulation of IFNγ signaling was associated with LC and correlated with signatures of reduced T cell activation and increased T cell exhaustion, suggesting that chronic immune stimulation may lead to functional impairment of T cells. These findings are consistent with prior observations^5,28^ and suggest the potential role of T cell dysregulation and exhaustion in LC pathogenesis.

Our study also confirms and extends prior reports of metabolic dysregulation in LC^1,8,10,12,19,22,26,30,33^. We observed a decrease in amino acid metabolism and an increase in corticotropin-releasing hormone signaling, leptin signaling, fatty acid metabolism, bile acid and beta-alanine metabolism in LC. Moreover, these metabolic pathways correlated with proinflammatory pathways in the LC group, suggesting a link between metabolic dysregulation and chronic inflammation. We also observed decreased activity of the telomere maintenance and DNA damage recognition and repair pathways, chromatin regulation and DNA methylation in the LC group. Impaired telomere maintenance could be associated with premature cellular senescence or apoptosis that may impede tissue repair processes^45,46^.

Our study is limited by relatively small cohorts of individuals with LC who were predominantly female and with symptom clusters that primarily involved fatigue, brain fog and pain. Larger studies from more diverse populations will be required to assess the generalizability of our findings. Nevertheless, we observed good concordance between the 2020–2021 initial cohort and the 2023–2024 validation cohort. Another limitation is the use of bulk RNA-seq, which limits more detailed resolution of pathways at the cellular level. Therefore, future studies should use single-cell transcriptomic and T cell profiling technologies to provide higher-resolution data. Nevertheless, our observations suggest potential therapeutic targets for LC that could be explored in clinical trials. Because the IL-6 and JAK-STAT pathways were among the top upregulated pathways in participants with LC in both the 2020–2021 and the 2023–2024 cohorts, we have initiated a clinical trial to evaluate the therapeutic efficacy of the JAK1 inhibitor abrocitinib for LC (NCT06597396).

In conclusion, our data demonstrate that LC is characterized by chronic inflammation, immune exhaustion and metabolic dysregulation. Current therapeutic efforts are largely focused on antiviral agents to address potential residual viral replication. However, the lack of efficacy of nirmatrelvir-ritonavir in treating LC highlights the need to explore alternative therapeutic strategies^34^. Our data suggests that the JAK-STAT and IL-6 pathways, and the IFN and metabolic pathways, are potential therapeutic targets that could be evaluated for LC.

## Methods

### Clinical cohorts

The 2020–2021 cohort samples were collected between April 2020 and October 2021 as part of the MassCPR COVID-19 biorepository. This discovery cohort of 142 individuals included UCs (*n* = 35, 51% female and 49% male), acutely infected individuals (*n* = 54, 50% female and 50% male), CCs (*n* = 24, 60% female and 40% male) and patients with LC (*n* = 28, 86% female and 14% male). Samples were collected during the acute phase (< 30 days after infection) and during the chronic phase between 30 and 300 days after infection.

The 2023–2024 validation cohort samples were collected between October 2022 and December 2024 as part of the BIDMC biorepository jointly with the BIDMC clinical site of the NIH RECOVER study with the complete clinical meta-data and structured symptom questionnaires. This validation cohort of 38 individuals included CCs (*n* = 20, 55% female and 45% male) and patients with LC (*n* = 18, 78% female and 22% male). Samples were collected during the acute phase (< 30 days after infection) and during the chronic phase between 30 and 700 days after infection.

Symptoms in the 2020–2021 cohort were self-reported, whereas symptoms in the 2023–2024 cohort were collected with the RECOVER standardized symptom questionnaire. All samples were collected as part of the MassCPR, RECOVER and BIDMC biorepository studies with institutional review board approval and participant informed written consent. De-identified and cryopreserved samples were used in this study.

### NAb assay

The nAb titers against the SARS-CoV-2 variants were determined using pseudotyped viruses expressing a LUC reporter gene. In brief, a LUC reporter plasmid pLenti-CMV Puro-Luc (Addgene), the packaging construct psPAX2 (AIDS Resource and Reagent Program) and Spike protein expressing pcDNA3.1-SARS-CoV-2 SΔCT were cotransfected into human HEK 293T cells (CRL-3216, ATCC) with lipofectamine 2000 (Thermo Fisher Scientific). Pseudotyped viruses of the SARS-CoV-2 variants were generated using the Spike protein from WA1/2020 (Wuhan/WIV04/2019, GISAID accession ID: EPI_ISL_402124), B.1.617.2 (Delta, GISAID accession ID: EPI_ISL_2020950) and Omicron BA.1 (GISAID accession ID: EPI_ISL_7358094.2). Forty-eight hours after transfection, the supernatants containing the pseudotyped viruses were collected and purified using filtration with 0.45-μm filter. To determine nAb titers in human sera, HEK-293T-hACE2 cells were seeded in 96-well tissue culture plates at a density of 2 × 10^4^ cells per well overnight. Three-fold serial dilutions of heat-inactivated serum samples were prepared and mixed with 60 μl of pseudovirus and incubated at 37 °C for 1 h before adding to the HEK-293T-hACE2 cells. Forty-eight hours later, cells were lysed in Steady-Glo Luciferase Assay (Promega Corporation) according to the manufacturer’s instructions. SARS-CoV-2 neutralization titers were defined as the sample dilution at which a 50% reduction (NT_50_) in relative light units was observed relative to the average of the virus control wells. Titers greater than 1:20 were considered positive.

### IFNγ ELISpot assay

Cellular immune responses specific to SARS-CoV-2 were assessed using IFNγ ELISpot assays using pools of overlapping 15-amino-acid peptides for the Wisconsin delta and omicron variants (21st Century Biochemicals). Ninety-six-well multiscreen plates (Merck Millipore) were coated with 1 µg per well of mouse antihuman IFNγ (MabTech) overnight in endotoxin-free Dulbecco’s PBS (DPBS) overnight at 4 °C. Plates were washed with DPBS three times and blocked using Roswell Park Memorial Institute 1640 media containing 10% FCS for 2–4 h at 37 °C. Peptides pools were prepared at a concentration of 2 µg per well, and 200,000 cells per well were added. Peptides and cells were incubated for 15–20 h at 37 °C. The plates were washed with DPBS-Tween seven times and then incubated with 1 µg ml^−1^ per well of biotinylated antihuman IFNγ (MabTech) for 2–4 h at room temperature, followed by four washes with DPBS-Tween and 1.33 µg ml^−1^ per well of alkaline phosphatase-conjugated anti-biotin (Rockland) for 2–3 h at room temperature. Plates were developed with nitroblue tetrazolium-5-bromo-4-chloro-3-indolyl-phosphate chromogen (Pierce), stopped by washing with tap water, and read using an ELISpot reader (KS ELISPOT Reader, Carl Zeiss). The number of spot-forming cells per 10^6^ cells were calculated, subtracted over background (PBMCs incubated with medium and dimethylsulfoxide without peptide).

### Bulk RNA-seq

PBMCs were lysed in 700 μl of TRIzol and then extracted using the miRNeasy Mini Kit (QIAGEN) with on-column DNase digestion. RNA quality was assessed using a TapeStation 4200 (Agilent Technologies) and then 10 ng of total RNA was used as input for complementary DNA (cDNA) synthesis using the Clontech SMART-Seq v4 Ultra Low Input RNA Kit (Takara Bio) according to the manufacturer’s instructions. Amplified cDNA was fragmented and appended with dual-indexed barcodes using the Nextera XT DNA Library Preparation Kit (Illumina). Libraries were validated using capillary electrophoresis on a TapeStation 4200, pooled at equimolar concentrations and sequenced with PE100 reads on an Illumina NovaSeq 6000, yielding ~30 million reads per sample on average. Alignment was performed using STAR v.2.7.3a^47^; transcripts were annotated using a composite genome reference that included the GRCh38 Ensembl release 100 and SARS-CoV-2 (GCF_009858895.2, ASM985889v3, MN985325.1). Transcript abundance estimates were calculated internal to the STAR aligner using the htseq-count algorithm. Transcript abundance estimates were calculated internal to the STAR aligner using the htseq-count algorithm. DESeq2 (ref. ^48^) was used for normalization, producing a normalized read count.

To assess for viral transcription, sample reads were aligned to a reference of 59 complete viral genome sequences using the Burrows–Wheeler Aligner (v.0.7.17)^49^ to produce a sorted alignment (.bam) file. SAMtools (v.1.3.1) was used to summarize the number of reads aligned to each genome. The complete FASTA file is provided along with the bulk RNA-seq raw data.

### Plasma proteomics

A total of 55 ml serum or plasma from all participants, five pooled plasma controls and one buffer control were analyzed using the SomaScan Assay Kit for human plasma V4.1 (cat. no. 900-00020), measuring the expression of 6,596 unique human protein targets using 7,596 slow off-rate modified aptamer reagents (SOMAmer), single-stranded DNA aptamers, according to the manufacturer’s standard protocol (SomaLogic). The modified aptamer binding reagents, SomaScan assay, and its performance characteristics and specificity to human targets, have been described previously. The assay used standard controls, including 12 hybridization normalization control sequences used to control for variability in the Agilent microarray readout process, and five human calibrator control pooled plasma replicates and three quality control (QC) pooled replicates used to mitigate batch effects and verify the quality of the assay run using standard acceptance criteria. The readout was performed using the Agilent microarray hybridization, scan and feature extraction technology.

Twelve hybridization control SOMAmers were added alongside SOMAmers to be measured from the serum samples and controls of each well during the SOMAmer elution step to control for readout variability. The control samples were run repeatedly during assay qualification and robust point estimates were generated and stored as references for each SOMAmer result for the Calibrator and QC samples. The results are used as references for the SomaScan v.4.1 Assay. Plate calibration was performed by calculating the ratio of the calibrator reference relative fluorescence unit (RFU) value to the plate-specific calibrator replicate median RFU value for each SOMAmer. The resulting ratio distribution was decomposed into a plate scale factor defined by the median of the distribution and a vector of SOMAmer-specific calibration scale factors. Normalization of QC replicates and samples was performed using adaptive normalization by maximum likelihood with point and variance estimates from a normal US population. Post-calibration accuracy was estimated using the ratio of the QC reference RFU value to the plate-specific QC replicate median RFU value for each SOMAmer. The resulting QC ratio distribution provides a robust estimate of accuracy for each SOMAmer on every plate. SomaScan RFU values and clinical information were obfuscated to protect personally identifiable information while preserving biologically relevant biomarkers. These reference datasets were provided by SomaLogic. We used the limma R package to identify differentially expressed proteins in the LC and CC groups. The method involves fitting a linear model to the data and then performing a *t*-test to identify proteins that are differentially expressed between two or more groups. *P* values were corrected for multiple testing using the Benjamini–Hochberg method. The R packages ggplot2 and ComplexHeatmap were used to generated the figures. Pathway enrichment analysis was performed using GSEA (https://www.gsea-msigdb.org/gsea). Genes were preranked according to the fold change from the highest to the lowest; GSEA was used to assess the enrichment of selected gene sets. Cytokine signaling, immune cell signatures and molecular pathways were compiled from the MSigDB Hallmark, C2, C7 and C3 gene sets (www.gsea-msigdb.org/gsea/msigdb/collections.jsp) and the blood transcriptional modules. The GSEA Java desktop program was downloaded from the Broad Institute (www.broadinstitute.org/gsea/index.jsp) and used with GSEA preranked module parameters (number of permutations: 1,000; enrichment statistic: weighted; 10 ≤ gene set size ≤ 5,000)^50^. Sample-level enrichment analysis^50^ was used to investigate the enrichment of pathways in each animal. Briefly, the expression of all genes in a specific pathway was averaged across samples and compared to the average expression of 1,000 randomly generated gene sets of the same size. The resulting *z*-score was then used to reflect the overall perturbation of each pathway in each sample.

### ssGSEA

We estimated pathway activity per sample using ssGSEA^51^. Leading-edge gene lists were parsed from the prior GSEA output and used as the gene sets for each pathway. For every sample, genes were ranked according to normalized expression and an enrichment score was computed as the difference between the empirical cumulative distributions of ranks for leading-edge genes versus all other genes; higher scores indicate greater pathway activation. Scores were normalized (per sample and then *z*-scored across samples) to enable comparisons, yielding a samples × pathways ssGSEA matrix for the downstream analyses.

### Gene expression correlation analysis between symptoms and pathway scores

To explore associations between symptom presence and gene expression signatures, we merged the one-hot symptom mapping with the original cohort data containing pathway-level gene expression profiles. For each of the 18 symptom categories, we then identified all patients who had that symptom and averaged their gene expression values across all measured pathways. In other words, for each symptom like fatigue or brain fog, we looked at all the patients who had that symptom and calculated the average expression level of each gene pathway in that subgroup. This produced an 18 × *n* matrix (where *n* is the number of gene pathways), with each cell representing the average expression in patients with that symptom. The resulting correlation matrix was visualized using a heatmap with color mapping reflecting average expression levels. This can be formally represented as:

$${C}_{j,k}=\frac{1}{|{S}_{j}|}\mathop{\sum }\limits_{i\in {Sj}}{G}_{i,k}$$

Where *C*_*j,k*_ is the average expression of gene pathway (*k*) for symptom (*j*), *S*_*j*_ is the set of patients with symptom (*j*) and *G*_*i,k*_ is the expression value of gene pathway (*k*) for patient (*i*).

### ELISA

Cytokines were assessed using ELISA. An antihuman cytokine coating antibody was adsorbed onto 96-microwell plates. Microwells were washed with wash buffer; human serum samples and human cytokine standards were prediluted in assay buffer and added to each plate. Plates were then incubated for 1 h before adding antihuman cytokine horseradish peroxidase (HRP). After an additional 1-h incubation, plates were again washed with wash buffer. SeraCare KPL TMB SureBlue Start solution was added to each well; plate development was halted by adding SeraCare KPL TMB Stop solution to each well. Absorbance at 450 nm was recorded with a VersaMax Microplate Reader (Molecular Devices). The standard curve was prepared from human cytokine standard dilutions and the human cytokine concentration was determined. For each sample, the cytokine concentration was calculated using a four-parameter logistic curve fit; cytokine concentrations of the unknown samples were interpolated from the linear portion of the standard curve generated from the human cytokine standards of known concentration.

### MSD

Serum levels of human IL-6R were tested using the R-PLEX Human IL-6R Kits from Meso Scale Discovery (cat. no. K1510GR-2) by the Metabolism and Mitochondrial Research Core (BIDM) according to the manufacturer’s instructions. Briefly, the plate was coated using the provided biotinylated capture antibody (1:100 dilution). The highest Calibrator standard is 2,000 pg ml^−1^. Then a fourfold serial dilution was done to generate seven calibrator curve using a four-parameter logistic model. The detection limit is 0.4 pg ml^−1^. Samples were thawed on ice and diluted at a 1:200 ratio using the Diluent 7 provided by the kit. The assay plate was read by a MESO QuickPlex SQ 120 instrument and data were analyzed using the Discovery workbench 4.0 software.

### RF analysis

We applied ssGSEA, which transforms gene expression profiles into enrichment scores for predefined gene sets (for example, pathways), thereby producing a matrix in which each row corresponds to a sample and each column represents a pathway activity score. To assess the contribution of individual pathways, we trained an RF classifier using the ssGSEA scores as input features. The dataset was randomly split into a training set (70%) and a testing set (30%) using stratified sampling to preserve the class distribution. The RF model was trained with 500 trees using the randomForest R package, where the outcome variable was CLASS (LC or CC) and the predictors were the ssGSEA-derived pathway scores. Model performance was evaluated using the held-out test set, with classification accuracy and a confusion matrix computed to assess prediction quality. To determine the relative importance of each pathway in predicting the LC outcome, we extracted the mean decrease in Gini impurity (MDG) from the trained RF model. The MDG quantifies how much each feature (pathway/gene) contributes to reducing classification uncertainty. A scatter plot was created to display the relationship between prediction accuracy and MDG for each pathway using transcriptomic and proteomic pathways. We added bar plots to visualize the top pathways/genes ranked according to importance generated by the RF model. Each bar represents a pathway; its length reflects its importance in the RF model. This plot highlights the most influential pathways in distinguishing LC from CC based on ssGSEA scores. All figures were generated using R v.4.4.2, using standard visualization packages for statistical and transcriptomic data analysis.

### Statistical analyses

The data from the RNA-seq and proteomics were analyzed with RStudio (v.4.4.0) in PRISM 9.0 (GraphPad Software). Pathways enrichment analysis was performed using the GSEA software. Sample-level ssGSEA was performed using the GSVA (v.1.40) R package. The ssGSEA analysis delineates a comparative exploration of the groups’ median values, visually represented through the data distribution and median lines. Statistical significance, denoted by the calculated *P* value, is highlighted above the comparison line, where a *P* < 0.05 is considered statistically significant. The two-sided Mann–Whitney *U*-test was used to identify significant differences between groups. For more than one group comparison, we used a Kruskal–Wallis test to compare all three groups; when significant, we performed post hoc pairwise comparisons with the Dunn’s test with Bonferroni correction for multiple comparisons. nAb titers and IFNγ ELISpot counts were analyzed after log_10_ transformation; to avoid undefined values, measurements <1 were set to 1 before transformation. For each viral variant (WH/2020, Delta, BA.1), CC and LC groups were compared using a two-sided Wilcoxon rank-sum (Mann–Whitney *U*-test) test on the log_10_ values. Multiple testing across variants within each assay (nAb and ELISpot analyzed separately) was controlled using the Benjamini–Hochberg procedure; adjusted *q*values are reported in the subpanel titles; nominal *P* values are shown above the parentheses. A nonparametric test was selected a priori because immune readouts are typically right-skewed with potential outliers and unequal variances, and the groups are independent. Individual data and group medians are displayed in the plots. Analyses were performed in R using the base wilcox.test with Benjamini–Hochberg adjustment. Because of the limited sample availability, no statistical methods were used to predetermine sample sizes and we used all the samples that were made available to us. Where applicable, data distribution was assumed to be normal but this was not formally tested.

### Reporting summary

Further information on research design is available in the Nature Portfolio Reporting Summary linked to this article.

## Online content

Any methods, additional references, Nature Portfolio reporting summaries, source data, extended data, supplementary information, acknowledgements, peer review information; details of author contributions and competing interests; and statements of data and code availability are available at 10.1038/s41590-025-02353-x.

## Supplementary information

Reporting Summary

Supplementary Table 1

Supplementary Table 2

Supplementary Table 3

## Data Availability

All data are available in the manuscript or the supplementary materials. Transcriptomic raw data have been deposited in the Gene Expression Omnibus under accession no. GSE226260.

## Code availability

The analysis code, data preparation, stratified subject-level splitting, RF training and ROC/AUC evaluation are available at https://github.com/Barouch-and-BCGE-Labs/PASC-Project.
