# Author Correction: Long COVID involves activation of proinflammatory and immune exhaustion pathways

**DOI:** 10.1038/s41590-026-02438-1

**Published:** 2026-01-23

**Authors:** Malika Aid, Valentin Boero-Teyssier, Katherine McMahan, Rammy Dang, Michael Doyle, Nazim Belabbaci, Erica Borducchi, Ai-ris Y. Collier, Janet Mullington, Dan H. Barouch

**Affiliations:** 1 Center for Virology and Vaccine Research, Beth Israel Deaconess Medical Center, Boston, MA USA; 2 Division of Sleep Medicine, Beth Israel Deaconess Medical Center, Boston, MA USA

**Keywords:** Viral infection, SARS-CoV-2

Correction to: *Nature Immunology* 10.1038/s41590-025-02353-x, published online 12 December 2025.

In the version of the article initially published, Rammy Dang’s surname appeared incorrectly (as Dong) and has now been corrected in the HTML and PDF versions of the article.

