# Supplementary material for: Long COVID involves activation of proinflammatory and immune exhaustion pathways
Source: Nat Immunol. 2025 Dec 12;27(1):61–71. doi: 10.1038/s41590-025-02353-x (PMC12764429; doi:10.1038/s41590-025-02353-x)
Supplement: Supplementary file 1 — Reporting Summary [file 41590_2025_2353_MOESM1_ESM.pdf]

nature portfolio

Corresponding author(s): Dan Barouch

Last updated by author(s): Oct 21, 2025

Reporting Summary

Nature Portfolio wishes to improve the reproducibility of the work that we publish. This form provides structure for consistency and transparency in reporting. For further information on Nature Portfolio policies, see our Editorial Policies and the Editorial Policy Checklist.

Statistics

For all statistical analyses, confirm that the following items are present in the figure legend, table legend, main text, or Methods section.

n/a	Confirmed	
☐	☒	The exact sample size (*n*) for each experimental group/condition, given as a discrete number and unit of measurement
☐	☒	A statement on whether measurements were taken from distinct samples or whether the same sample was measured repeatedly
☐	☒	The statistical test(s) used AND whether they are one- or two-sided<br>*Only common tests should be described solely by name; describe more complex techniques in the Methods section.*
☒	☐	A description of all covariates tested
☐	☒	A description of any assumptions or corrections, such as tests of normality and adjustment for multiple comparisons
☐	☒	A full description of the statistical parameters including central tendency (e.g. means) or other basic estimates (e.g. regression coefficient) AND variation (e.g. standard deviation) or associated estimates of uncertainty (e.g. confidence intervals)
☐	☒	For null hypothesis testing, the test statistic (e.g. *F*, *t*, *r*) with confidence intervals, effect sizes, degrees of freedom and *P* value noted<br>*Give P values as exact values whenever suitable.*
☒	☐	For Bayesian analysis, information on the choice of priors and Markov chain Monte Carlo settings
☒	☐	For hierarchical and complex designs, identification of the appropriate level for tests and full reporting of outcomes
☒	☐	Estimates of effect sizes (e.g. Cohen's *d*, Pearson's *r*), indicating how they were calculated

Our web collection on statistics for biologists contains articles on many of the points above.

Software and code

Policy information about availability of computer code

Data collection	N/A
Data analysis	Alignment was performed using STAR version 2.7.3a; DESeq2 was used for normalization, producing a normalized read count; the following tools were used to estimate viral read counts : Burrows-Wheeler Aligner (bwa 0.7.17) and Samtools (v1.3.1) . P values were corrected for multiple testing using the Benjamini-Hochberg method. Pathways analysis was in R.4.4.0 using the following packages : GSEA, SLEA, and GSVA (v1.40). Statistical analysis was performed in R and in Graphpad PRISM 9.0 software (GraphPad Software, San Diego, California, USA). Machine learning was performed using the Random forest method in R :randomForest 4.7-1.2.

For manuscripts utilizing custom algorithms or software that are central to the research but not yet described in published literature, software must be made available to editors and reviewers. We strongly encourage code deposition in a community repository (e.g. GitHub). See the Nature Portfolio guidelines for submitting code & software for further information.

Data

Policy information about availability of data

All manuscripts must include a data availability statement. This statement should provide the following information, where applicable:

- Accession codes, unique identifiers, or web links for publicly available datasets
- A description of any restrictions on data availability
- For clinical datasets or third party data, please ensure that the statement adheres to our policy

All data are available in the manuscript or the supplementary material. Bulk RNA-sequencing raw data has been deposited in the GEO database under accession number GSE226260.

Research involving human participants, their data, or biological material

Policy information about studies with human participants or human data. See also policy information about sex, gender (identity/presentation), and sexual orientation and race, ethnicity and racism.

Reporting on sex and gender	This study did not include a sex/gender analysis due to the limited sample size that did not allow us to conduct a detailed and separate analysis based on gender or sex.
Reporting on race, ethnicity, or other socially relevant groupings	N/A
Population characteristics	N/A
Recruitment	The 2020-2021 cohort samples were collected between April 2020 and October 2021 as part of the Massachusetts Consortium for Pathogen Readiness (MassCPR) COVID-19 biorepository. This discovery cohort of 142 individuals included uninfected controls (N=35, 51% female and 49% male), acutely infected individuals (N=54, 50% female and 50% male), convalescent controls (N=24, 60% female and 40% male), and long Covid patients (N=28, 86% female and 14% male). Samples were collected during the acute phase (<30 days after infection) and during the chronic phase between 30 to 300 days after infection.<br><br>The 2023-2024 validation cohort samples were collected between October 2022 and December 2024 as part of the Beth Israel Deaconess Medical Center (BIDMC) biorepository jointly with the BIDMC clinical site of the NIH RECOVER study with the complete clinical metadata and structured symptom questionnaires. This validation cohort of 38 individuals included convalescent controls (N=20, 55% female and 45% male) and long Covid patients (N=18, 78% female and 22% male). Samples were collected during the acute phase (<30 days after infection) and during the chronic phase between 30 to 700 days after infection.<br><br>Symptoms in the 2020-2021 cohort were self-reported, whereas the symptoms in the 2023-2024 cohort were collected with the RECOVER standardized symptom questionnaire.
Ethics oversight	The Massachusetts Consortium for Pathogen Readiness (MassCPR) COVID-19 biorepository and the NIH RECOVER prospective clinical trial study

Note that full information on the approval of the study protocol must also be provided in the manuscript.

Field-specific reporting

Please select the one below that is the best fit for your research. If you are not sure, read the appropriate sections before making your selection.

☒ Life sciences ☐ Behavioural & social sciences ☐ Ecological, evolutionary & environmental sciences

For a reference copy of the document with all sections, see nature.com/documents/nr-reporting-summary-flat.pdf

Life sciences study design

All studies must disclose on these points even when the disclosure is negative.

Sample size	Samples were made available for us by the The Massachusetts Consortium for Pathogen Readiness (MassCPR) COVID-19 biorepository and the NIH RECOVER prospective clinical trial study , thus we did not perform any power/size effect analysis prior to selecting samplers.
Data exclusions	No data were excluded from this study
Replication	We did not perform replicates within each cohort due limited samples , however, we conducted the same analysis in two independent cohorts to validate our findings.

Randomization	N/A
Blinding	N/A

Reporting for specific materials, systems and methods

We require information from authors about some types of materials, experimental systems and methods used in many studies. Here, indicate whether each material, system or method listed is relevant to your study. If you are not sure if a list item applies to your research, read the appropriate section before selecting a response.

Materials & experimental systems

n/a	Involved in the study
☒	☐ Antibodies
☒	☐ Eukaryotic cell lines
☒	☐ Palaeontology and archaeology
☒	☐ Animals and other organisms
☐	☒ Clinical data
☒	☐ Dual use research of concern
☒	☐ Plants

Methods

n/a	Involved in the study
☒	☐ ChIP-seq
☒	☐ Flow cytometry
☒	☐ MRI-based neuroimaging

Clinical data

Policy information about clinical studies

All manuscripts should comply with the ICMJE guidelines for publication of clinical research and a completed CONSORT checklist must be included with all submissions.

Clinical trial registration	Clinical sample collections were performed as part of other studies. De-identified and cryopreserved samples were used in this study.
Study protocol	N/A
Data collection	N/A
Outcomes	N/A

Plants

Seed stocks	N/A
Novel plant genotypes	N/A
Authentication	N/A
